# Development and marker-trait relationships of functional markers for glutamine synthetase *GS1* and *GS2* homoeogenes in bread wheat

**DOI:** 10.1007/s11032-022-01354-0

**Published:** 2023-01-19

**Authors:** Laura Pascual, Aida Solé-Medina, Isabel Faci, Patricia Giraldo, Magdalena Ruiz, Elena Benavente

**Affiliations:** 1 Department of Biotechnology-Plant Biology, School of Agricultural, Food and Biosystems Engineering, Universidad Politécnica de Madrid, 28040 Madrid, Spain; 2 Department of Forest Ecology & Genetics, Forest Research Centre (INIA, CSIC), Ctra. de La Coruña Km 7.5, 28040 Madrid, Spain; 3 John Innes Centre, Norwich Research Park, Colney, NR4 7UH UK; 4 Instituto Nacional de Investigación y Tecnología Agraria y Alimentaria (INIA), CSIC, Autovía A2, Km. 36.2, Finca La Canaleja, Alcalá de Henares, Madrid, 28805 Spain

**Keywords:** *Triticum aestivum*, N-use efficiency, Marker-assisted selection, Epistatic interactions, Gene pyramiding

## Abstract

*GS1* and *GS2* genes encode, respectively, the main cytosolic and the plastidic isoforms of glutamine synthetase (GS). In the present study, the wheat *GS1* and *GS2* homoeogenes located in the A, B and D genome chromosomes have been sequenced in a group of 15 bread wheat varieties including landraces, old commercial varieties and modern cultivars. Phenotypic characterization by multi-environment field trials detected significant effects of specific GS homoeogenes on three of the seven agronomic and grain quality traits analyzed. Based on the gene sequence polymorphisms found, biallelic molecular markers that could facilitate marker-assisted breeding were developed for genes *GS1A*, *GS2A* and *GS2D*. The remaining genes encoding main wheat GS were excluded because of being monomorphic (*GS1D*) or too polymorphic (*GS1B* and *GS2B*) in the sequencing panel varieties. A collection of 187 Spanish bread wheat landraces was genotyped for these gene-based molecular markers. Data analyses conducted with phenotypic records reported for this germplasm collection in López-Fernández et al. (Plants-Basel 10: 620, 2021) have revealed the beneficial influence of some individual alleles on thousand-kernel weight (TKW), kernels per spike (KS) and grain protein content. Furthermore, genetic interactions between *GS1A*, a cytosolic GS isoform coding gene, and *GS2A* or *GS2D*, plastidic GS enzyme coding genes, were found to affect TKW and KS. The finding that some alleles at one locus may mask the effect of positive alleles at hypostatic GS loci should be kept in mind if gene pyramiding strategies are attempted for the improvement of N-use efficiency-related traits.

**Supplementary Information:**

The online version contains supplementary material available at 10.1007/s11032-022-01354-0.

## Introduction

Improvement of N-use efficiency (NUE) is a current critical issue for plant breeding in order to maximize crop production with less harmful environmental consequences. Recent review papers have dealt with the genetic basis of this complex trait (Cormier et al. 2016; Islam et al. 2021; Liu et al. 2022; Teng et al. 2022) whose relatively low genetic progress in the past decades may reflect an indirect effect of breeding programmes oriented to increase grain yield at standard high input cropping practices (Cormier et al. 2016). Active selection for efficient N uptake or utilization seems then an urgent breeding strategy to create novel crop cultivars that can respond to the increasing global food demand in more sustainable agroecosystems. Furthermore, in the case of wheat, NUE is not only relevant for grain yield but also for grain quality and flour or semolina industrial processing properties (Laidig et al. 2022; OrtizMonasterio et al. 1997).

Glutamine synthetase (GS) catalyses the synthesis of the amino acid glutamine, the first step by which plants incorporate inorganic N into an organic molecule, thus having a key role in the assimilation and mobilization of N to the target plant organs (e. g., Liu et al. 2022; Masclaux-Daubresse et al. 2010; Miflin and Habash 2002). Several GS isoforms have been chemically and functionally described, the most widely characterized being the cytosolic GS1 and the plastidic GS2 (Thomsen et al. 2014; Tobin et al. 1985; Unno et al. 2006; Wang et al. 2015). In common wheat, GS1 is encoded by three genes (hereafter referred to as *GS1A*, *GS1B* and *GS1D*) located on the homoeologous group-6 chromosomes, while the plastidic enzyme is controlled by three genes (hereafter referred to as *GS2A*, *GS2B* and *GS2D*) located on the homoeologous group-2 chromosomes (Habash et al. 2007). Two additional cytoplasmic isoforms, GSr and GSe, are each encoded by two homoeogenes located on chromosomes 4A and 4B (Gadaleta et al. 2014; Habash et al. 2007).

Many studies have aimed to relate the level of activity of GS enzymes with grain characteristics that are involved in yield performance or end-use quality of wheat cultivars, with a special focus on thousand kernel weight (TKW) and grain protein content (GPC) (e.g., Fontaine et al. 2009; Habash et al. 2007; Nigro et al. 2016). The role of GS activity in plant response to deprived fertilizer regimes has been widely examined, and the relationship between GS activity and crop performance under drought, heat or salinity stress has also been explored (e.g., Jallouli et al. 2019; Kichey et al. 2006; Nagy et al. 2013). In a few instances, the enzymatic activity of specific isoforms has been determined, making it possible to ascribe the effects eventually detected to the expression of a particular GS gene family at the protein level (Habash et al. 2001; Thomsen et al. 2014). After cloning of *GS1* and *GS2* wheat homoeogenes by Bernard et al. (2008), transcriptomic analyses have allowed to further characterize the expression at the RNA level of separate GS gene families and, in some instances, of specific *GS1* or *GS2* genes (e.g., Gayatri et al. 2021; Wei et al. 2020, 2021; Yousfi et al. 2016; Zhang et al. 2017a, b).

Changes in the expression of *GS1* and *GS2* genes between plant organs, and during plant development, have been reported either at the protein or RNA level (Bernard et al. 2008; Habash et al. 2001; Kichey et al. 2006; Zhang et al. 2017b, c). Overall, it has been showed that the expression of *GS2* declines from leaf vegetative development to leaf senescence and grain filling stage, when *GS1* expression seems relatively more relevant. This has led to suggest that GS2 activity could be mainly associated with traits eventually determined at the pre-anthesis stages (i.e., number of spikes per plant or kernels per spike), GS1 activity having a greater influence on traits which depend on N remobilization during grain development (Bernard et al. 2008; Habash et al. 2001; Masclaux-Daubresse et al. 2010).

In addition to the influence of external conditions and developmental stage of wheat plants on GS activity, genotype effects are also widely demonstrated (Fatholahi et al. 2020; Fontaine et al. 2009; Nigro et al. 2016). However, the studies that have attempted to link specific genotype variation at GS loci with plant traits assumed to be affected by GS activity are relatively scarce. The earliest among them conducted quantitative genetic analyses to determine the mapping position of QTLs influencing GS activity and, subsequently, their eventual colocalization with QTLs associated to agronomic traits (Fontaine et al. 2009; Habash et al. 2007). This indirect approach to identify allelic variants of GS genes with enhanced effect on NUE-related traits has been questioned not only because enzymatic activities, usually measured under controlled conditions, are highly dependent on the environment but also because genotype-by-environment effects are common on physiological traits (Fontaine et al. 2009). Nevertheless, several of those indirect genetic mapping approaches have reported QTLs for NUE-related traits that colocalize with structural genes coding for GS1 and GS2 in wheat (see Cormier et al. 2016 and references therein).

From a practical breeding point of view, routine screening of early segregant populations can hardly be based on measurement of protein activity or gene expression level. Some studies have then aimed to directly associate allelic variation at individual *GS1* or *GS2* homoeogenes, or genetically linked QTLs, with agronomic or quality traits of interest for NUE improvement (Cui et al. 2016; Guo et al. 2013; Li et al. 2019, 2011); but their results, when contrastable, have not always been consistent. So, Guo et al. (2013) reported the beneficial effect on TKW of one *GS1* allele (erroneously mapped to chromosome 6D; see the Discussion section) in a RIL population. However, no significant effect of that allele was found in a wide collection of Chinese winter wheat cultivars where, by contrast, the allele previously associated to lower TKW was present in the entries with the highest TKW values (Li et al. 2019). Furthermore, based on the inconsistent effects of overexpressing *GS1*, the success of improving NUE by means of simply increasing GS1 activity has been questioned (Thomsen et al. 2014). Regarding the wheat homoeogenes coding for the plastidic GS isoform, Li et al. (2011) reported a *GS2A* allele (originally designated *TaGS2-A1b* and later renamed to *TaGS2-2Ab*) associated with higher TKW and GPC, whose transgenic overexpression increased yield and several yield components (Hu et al. 2018). Li et al. (2011) described also a *GS2D* allele, designated *TaGS2-D1a*, associated with higher grain weight. A positive effect of that allele on TKW has been pointed out in a later study (Cui et al. 2016). However, no clue can be found in the literature cited by these authors (Cui et al. 2014a, b) on the actual relationship between the putatively functional marker used in the study, namely *IN10*, and the wheat GS locus on chromosome 2D. In durum wheat, the relationship between genotype variation at *GS2* homoeogenes and GPC has been analysed (Gadaleta et al. 2011; Nigro et al. 2017). Nigro et al. (2020) have actually reported a functional marker for *GS2B* related with high GPC. To our knowledge, no other earlier studies than those already mentioned have reported association between NUE-related traits and specific alleles of *GS1* and *GS2* genes or functional markers in wheat.

[On this point, it can be noted that gene symbols for wheat glutamine synthetase genes are not yet officially catalogued (https://wheat.pw.usda.gov/GG3/wgc). Renaming of some wheat GS genes and alleles in later reports and the use of almost identical nomenclature for genes controlling grain size (for example, *TaGS1a*, *TaGS2-A1* or *TaGS-D1* in Khalid et al. 2019), makes it troublesome to contrast published results on the effects of glutamine synthetase genes from literature reports.]

NUE is a highly complex trait as dependent on varied plant physiological processes. Nevertheless, the relatively few enzymatic activities involved in N assimilation supports that pyramiding of a small number of favourable alleles may have a significant impact on this component of NUE enhancement (Cormier et al. 2016; Islam et al. 2021). The application for wheat improvement of the cumulated knowledge on the effects of GS activity on NUE-related traits requires identification of GS genes alleles responsible of favourable N-use phenotypes. But molecular markers that may assist their selection in early breeding progenies and facilitate prebreeding germplasm screening are also needed. With that in mind, the final objective of this study was to develop functional markers for glutamine synthetase *GS1* and *GS2* wheat homoeogenes. A primary goal was to determine their sequence polymorphisms in a panel of 15 bread wheat genotypes whose phenotypic variation for agronomic and quality traits was also characterized by multi-environment field trials. The subsequent goal was to study whether molecular markers based on these polymorphisms were associated with yield components and grain quality traits in a wide, diverse, germplasm collection of bread wheat landraces.

## Materials and methods

### Plant material

The present study has been conducted on two distinct sets of bread wheat genotypes: a sequencing panel and a diversity panel. The sequencing panel was composed of fifteen varieties of bread wheat (*Triticum aestivum* L.) widely cultivated in Spain, either in the past or the present (Table 1). This group of genotypes included six landraces or pre-Green Revolution varieties (collected or released before 1960), four old commercial cultivars (released between 1970 and 1985) and five modern cultivars (released after 1990). The diversity panel was formed by 187 Spanish bread wheat landraces that constituted the primary set from which the Spanish bread wheat core collection was developed (Pascual et al. 2020a). The genetic structure and genomic variability of this collection was reported in Pascual et al. (2020b) while its phenotypic characterization for some agronomic and quality traits was further described in López-Fernández et al. (2021).

**Table 1:** Varieties forming the sequencing panel. Their haplotypes for *GS1* and GS2 homoeogenes are indicated. In all cases, haplotype 1 corresponds to the gene sequence in the reference cultivar Chinese Spring

				*GS* gene haplotype					
VARIETY	Type	Developer (a)	Release/ Collection date	*GS1A*	*GS1B*	*GS1D*	*GS2A*	*GS2B*	*GS2D*
NOGAL	commercial	Florimond Desprez	2006	2	2	1	1	3	2bis
ARTUR NICK	commercial	Limagrain	2002	2	1	1	2	4	1
CALIFA SUR	commercial	Limagrain	1999	1	1	1	1	1	1bis
BERDUN	commercial	Limagrain	1998	2	2	1	2	2	1
GAZUL	commercial	Limagrain	1992	3	1	1	2	2	2
ABLACA	commercial	Unknown	1982	1	1	1	2	7	1
MARIUS	commercial	Benoist	1980	2	2	1	2	6	2
ANZA	commercial	CIMMYT	1974	3	2	1	2	2	1
YECORA	commercial	CIMMYT	1972	1	1	1	1	1	1
PANE-247	commercial	Agrusa	1955	3	2	1	2	5	2
ARAGON-03 (b)	landrace	BGE012783	< 1940	2	2	1	2	1	1
CHAMORRO (b)	landrace	BGE012205	< 1940	2	3	1	2	1	1
MOCHO ROJO	local landrace	BGE012192	< 1940	2	3	1	2	8	1
CANDEAL VELLISCA	local landrace	BGE012591	< 1940	2	1	1	2	7	1
ROJO CARAVACA	local landrace	BGE018207	< 1940	2	4	1	2	2	1

(a) For landraces, the reference of the Spanish Germplasm bank of genetic resources is indicated; (b) Registered and currently commercialized

### DNA extraction and sequencing

All the accessions were germinated and DNA extraction was performed from 50 mg of coleoptile according to Doyle and Doyle (1990). The DNA was quantified in 1% agarose gels and stored at − 20 °C until used.

The sequence of the three homoeogenes coding for the cytosolic glutamine synthetase GS1 (*GS1A*, *GS1B* and *GS1D*), cloned by Bernard et al. (2008), and of the three homoeogenes coding for the plastidic glutamine synthetase (*GS2A*, *GS2B* and *GS2D*), cloned by Li et al. (2011), were blasted against the bread wheat reference genome (https://plants.ensembl.org/Triticum_aestivum/Tools/Blast). The obtained alignments were used to manually annotate the *GS* genes, determine the position of the UTRs and obtain the complete gene sequences including a 500 pb upstream and downstream window. From these sequences, primers were designed to cover the complete genomic sequence of each of the genes with three overlapping amplicons. The details of primers combinations and melting temperatures are given as supplementary material (Online Resource 1). PCR reactions were carried out with the DNA AmpliTools Complex Master Mix (Biotools, Madrid) following the manufacturer’s protocol.

The *GS1* and *GS2* amplicons were obtained for all the varieties included in the sequencing set. PCR amplified fragments were purified with sepharose columns and sequenced by capillary electrophoresis at Macrogen (Macrogen Europe, Amsterdam, The Netherlands). Sequences were analyzed with Sequencher® version 5.0 sequence analysis software (Gene Codes Corporation, Ann Arbor, MI, USA (http://www.genecodes.com) and aligned against the Chinese Spring sequence (IWGSC RefSeq v1.0) (International Wheat Genome Sequencing Consortium 2018). The obtained information was used to reconstruct the complete genomic sequence of all *GS1* and *GS2* homoeogenes and to define alleles (i.e., haplotypes). Nucleotide sequences for each identified allele were translated with ExPASy translation tool (http://web.expasy.org/translate/).

### Development of molecular markers and genotyping

Molecular markers were developed from the polymorphisms detected after sequencing the GS genes and employed to genotype the 187 varieties in the diversity panel. PCR markers were developed when the underlining polymorphism was an INDEL (*GS2A* and *GS2D*), and CAPS marker (Cleaved amplified polymorphic sequence) when the underling polymorphism was a SNP located within a restriction site (*GS1A*). For the CAPS marker, *GS1A* 2^nd^ amplicon was amplified as described in Online Resource 1 and PCR amplified fragments were digested with the restriction enzyme EcoRI (New England BioLabs Inc., MA, USA) following manufacturer’s protocol. Restriction fragments were then resolved on a 1% agarose gel. Regarding PCR markers, *GS2A* INDEL region was amplified with the pair of primers TTGATTGACTTCCATGAGAGCACA-Fw and TTAGCATAAAGCACGTCCAGATGA-Rev. For *GS2D* INDEL region, the primers employed to amplify the 2^nd^ amplicon of this gene were used (see Online Resource 1). The obtained amplicons were resolved on 1–1.5% agarose gels.

### Field trails and phenotype evaluation

The sequencing set of bread wheat varieties was evaluated in a total of six environments (i.e., site × year combinations). The trial design at La Canaleja (Alcalá de Henares; LC trials in 2014–2015, 2015–2016 and 2016–2017) consisted of three replicate plots (1.2 × 10 m in 2014–2015 and 2016–2017; 1.2 × 3 m in 2015–2016). In Limagrain’ station at Elorz (Navarra; LG trials in 2015–2016 and 2017–2018), the plots (1.5 × 8 m) were sown by duplicate. In both locations, completely randomized block designs were used. Finally, small plots (1 × 1 m) without replicate were established in the experimental fields of Universidad Politécnica de Madrid (Madrid; UPM trial in 2016–2017), the completely randomized design including two repeated control varieties. Fertiliser treatment in LC and UPM trials was 68 N units ha^−1^ in a single application. In LG trials, 70 N units ha^−1^ were split in two applications. Fungicides were not used in any of the trials. A post-emergency herbicide treatment was applied in LC and LG trials, metsulfuron-methyl being the active compound. No herbicide treatment was used in the UPM trial where weeds were controlled by manual weeding.

Summarized information on the experimental conditions and traits analysed in each trial is provided as supplementary information (Online Resource 2). Grain yield (GY, k ha^−1^ at 12% humidity) was estimated from grain weight at harvest in trials LC15, LC17, LG16 and LG18, while no GY estimation was attempted in trials with small plot sizes (LC16 and UPM17). After harvest, test weight (TW, kg hL^−1^) and thousand-kernel weight (TKW, g at 12% humidity) were measured for all accessions. Spike number (SN, m^−2^) was determined by extrapolating to 1 square meter the counting of the spikes in all plants contained in one-meter-long row. The number of kernels per spike (KS) was estimated as the mean number of kernels counted on 10 main spikes. Grain protein content (GPC, % on a dry matter basis) and gluten quality were evaluated on wholemeal flour samples. GPC was measured by near-infrared reflectance analysis using a PerCon Inframatic 8600 (Perten Instruments AB, Sweden). Gluten strength was estimated on 1 g of flour samples by the sodium dodecyl sulfate sedimentation volume (SVol, mm) test following the procedure of Dick and Quick (1983) with some modifications described in Chacon et al. (2020). All traits were analysed individually in each replicated plot, except SVol in LG trials where one flour sample per cultivar, obtained from a balanced mix of grains from the two replicates, was analysed. For GPC and SVol, technical duplicates were always prepared.

### Statistical analyses

Analyses of variance (ANOVA) were used to determine any significant effect of the replications in LC and LG trials. Once no effect was found, mean values of a given cultivar in each trial were used for further data analyses. The set of data for the agronomic and quality variables analysed in the diversity panel was taken from López-Fernández et al. (2021; provided there as supplementary information). Normality and homoscedasticity of variables was tested by the Shapiro–Wilk and Levene tests, respectively. For a better fit to normality, a logarithmic transformation was performed for KS before further analysis.

The influence of haplotypes or marker variants for *GS1* and *GS2* homoeogenes on the evaluated traits was studied by an analysis of variance using the general linear model (GLM) procedure, the significance threshold being set at *P* < 0.01. The GS genes, the environment (E) and GS gene × E interactions were used as sources of variation. In the sequencing panel, only haplotypes present in at least three cultivars were considered for analysis. In the diversity panel, interaction effects between GS genes were also estimated. Comparison of least squares means between alleles of a given GS gene, and between non-allelic variants combinations, was performed using Fisher’s protected least significant difference (LSD). Contingency chi-tests were used to check two-by-two independence between allelic variants at different GS loci in the diversity panel. All statistical analyses were completed with the InfoStat statistical package (Di Rienzo et al. 2020), which was also used for graphic representation of mean comparison analyses.

## Results

### Haplotyping of *GS1* and *GS2* homoeogenes

The sequences of *GS1* and *GS2* homoeogenes were determined and comparatively analysed in a panel of 15 wheat cultivars including local landraces, old cultivars and modern varieties (Table 1). Cultivar Chinese Spring was always used as reference for haplotype characterization.

For the *GS1A* gene, 12 SNPs and 3 INDELs were identified among the accessions analysed. The sequence variants allowed to define two new haplotypes (*GS1A-hap2* and *GS1A-hap3*) with respect to Chinese Spring (*GS1A-hap1*; see Online Resource 3). Two of the SNPs were located at the UTRs while the rest of polymorphisms affected at intronic regions. The most frequent allele (*GS1A-hap2*) was present in 9 cultivars including all the landraces. The other two haplotypes differed only in one SNP located in an intronic region, each of them being found in 3 varieties. At the gene *GS1B* we identified 13 SNPs and 3 INDELs, all except one (UTR) being located at intronic regions (see Online Resource 4). These polymorphisms were combined in a total of 4 alleles (*GS1B-hap1* to *4*). *GS1B-hap4*, only present in the local landrace Rojo Caravaca, was the most distant from the reference presenting a total of 15 polymorphisms. The other haplotypes differed only in one SNPs between them and were present in six (*GS1B-hap1*), six (*GS1B-hap2*) and two (*GS1B-hap3*) cultivars. No polymorphism was detected for gene *GS1D*, for which all 15 varieties presented the Chinese Spring reference sequence.

Sequence polymorphisms were detected for the three *GS2* homoeoloci (see Online Resources 5 to 7). For *GS2A*, a new allele (*GS2A-hap2*) presenting 8 SNPs and 5 INDELs with respect to Chinese Spring was identified. One of the polymorphisms was located in an UTR, another in an exon (synonymous) and the rest of them at intronic regions. *GS2A-hap2* was present in 12 cultivar including all the landraces, whereas the Chinese Spring allele (*GS2A-hap1*) was present in the remaining 3 cultivars. For *GS2B* we only obtained high quality sequence from 1200 pb downstream from the start codon to the end of the gene. In the sequenced region, which fully covered the 2^nd^ and 3^rd^ amplicons, we were able to detect a total of 9 SNPs and 1 INDEL. Two of the SNPs were located at exonic regions and one of them, located 1390 bp from the starting codon, produced a change in the coded protein (H163Q). This non-synonymous SNP was only present in cultivar Marius (*GS2B-hap6* allele). In total, we were able to identify 8 different alleles, the most frequent being the allelic variant present in Chinese Spring (*GS2B-hap1*, in 4 cultivars) and *GS2B-hap2* (4 cultivars). Finally, when we sequenced *GS2D* more than 50 polymorphisms were detected compared to the Chinese Spring reference. Thus, we decided to include in the alignment the *GS2D* sequence of cultivar Xiaoyan-54, reported by Li et al. (2011) (genebank accession no. GQ169689), and greatly differing from the Chinese Spring sequence either at the coding and 5’ flanking regions. Then, our cultivars were found to present either the Chinese Spring allele (*GS2D-hap1*) or the Xiaoyan-54 allele (here named *GS2D-hap2*), the only exceptions being two new SNPs. One of these changes was identified in cultivar Califa Sur (designated *GS2D-hap1bis*), that presented A instead of G at 72 bp from the start codon while no other polymorphism compared to Chinese Spring was detected for the rest of the gene. The other new SNP was detected in cultivar Nogal (designated *GS2D-hap2bis*), whose *GS2D* sequence was identical to *GS2D-hap2* except by a change A2190C (see Online Resource 7).

The haplotypes of the six GS genes under study in the varieties forming the sequencing panel are shown in Table 1. It can be highlighted that the five landraces in the set presented the same haplotypes for the *GS1* and *GS2* homoeogenes in the A and D genome, even though these haplotypes were also found in commercial varieties. However, some sequence variants of *GS1B* and *GS2B* were only found in the landraces (*GS1B-hap3* and *4*; *GS2B-hap8*).

### Field evaluation of the sequencing panel of bread wheat varieties

**Fig. 1:**
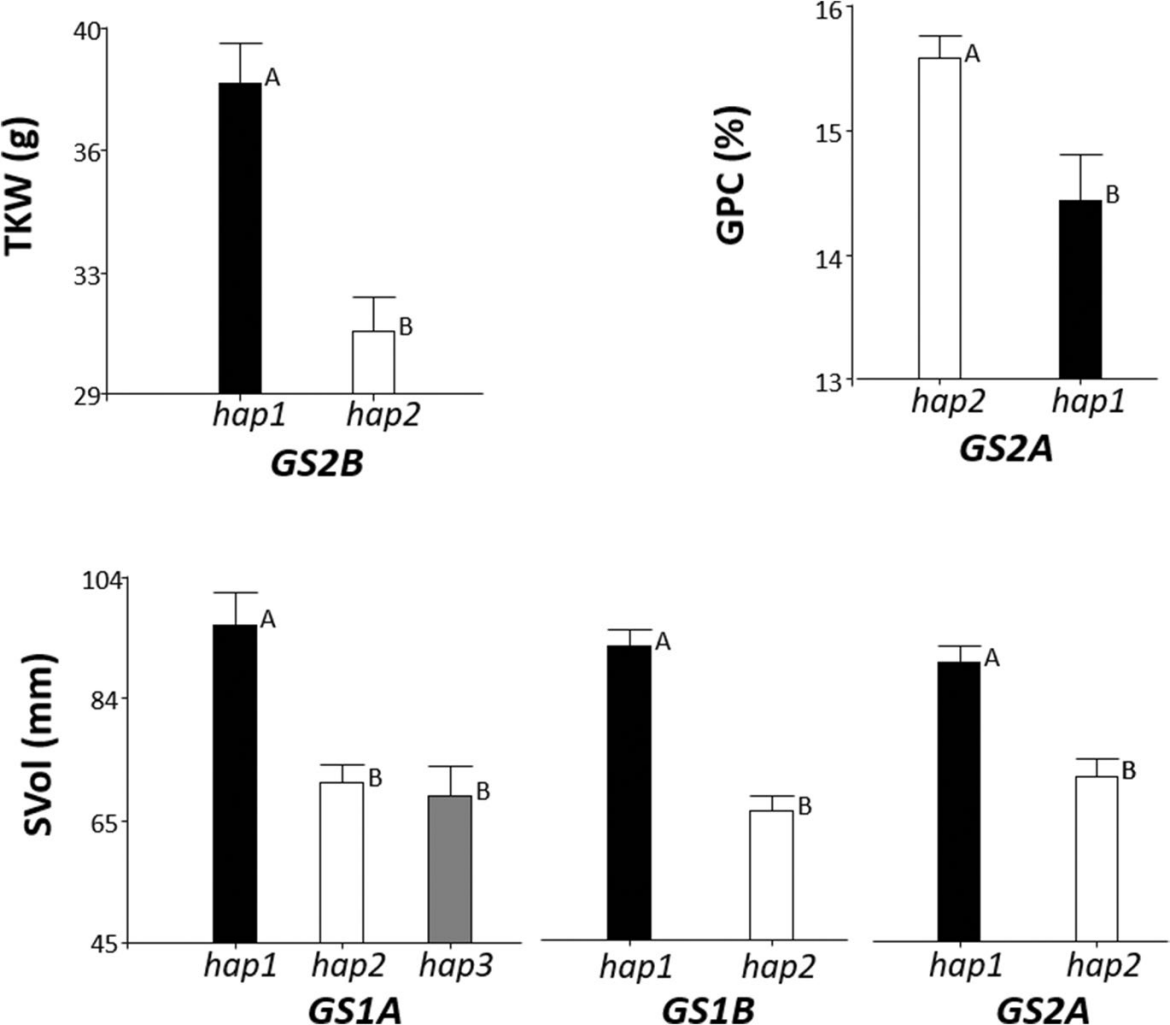
Mean phenotypic value associated to individual haplotypes for the *GS* loci with significant effect (*P* < 0.01) on the traits evaluated in the sequencing panel of wheat varieties. At each locus, the black bars indicate the Chinese Spring haplotype. Means with a common letter are not statistically different (*P* > 0.05). TKW: thousand-kernel weight; GPC: grain protein content; SVol: SDS-sedimentation volume

The data obtained for the seven agronomic and quality traits evaluated by field experiments on the sequencing panel of varieties are provided as supplementary information (Online Resource 8). The results of the analyses of variance conducted to determine the influence of environment and allelic variation at *GS1* and *GS2* homoeogenes are summarized in Online Resource 9. These analyses revealed that the environment was a highly significant source of variation for all parameters, except SVol. Figure 1 shows the mean phenotypic value associated to individual haplotypes for the GS loci with significant effect on the traits examined. As noted above, for each locus, only variants present in 3 or more accessions were considered for analysis.

None of the 5 GS genes under analysis had a significant influence on GY, TW, SN or KS (see Online Resource 9). Among the traits related to yield potential, only TKW was influenced by variation at a GS locus, the haplotype *GS2B-hap*1 being associated to heavier grains in the sequencing panel (Fig. 1). Grain protein content was found to be significantly affected by genotype variation at *GS2A*, *GS2A-hap2* being the haplotype with a positive effect on GPC. Finally, SVol, the unique trait for which environment effects were not detected, was found to be significantly influenced by genotype variation at *GS1A*, *GS1B* and *GS2A*.

### Molecular markers to discriminate *GS1* and *GS2* haplotypes

In order to develop GS gene-based molecular markers that could be useful for MAS in breeding programs, we focused on *GS1A*, *GS2A* and *GS2D*. For these three GS genes, the sequenced varieties either presented only two alleles or the additional alleles showed only one SNP compared to one of two clearly contrasting haplotypes. The latter occurred for *GS1A-hap3*, with regard to the Chinese Spring haplotype (*GS1A-hap1*), and for *GS2D-hap1bis* and *2bis* with regard to the Chinese Spring and Xiaoyan-54 haplotypes, *GS2D-hap1* and *GS2D-hap2*, respectively (Table 1; Online Resources 3, 5 and 7).

**Fig. 2:**
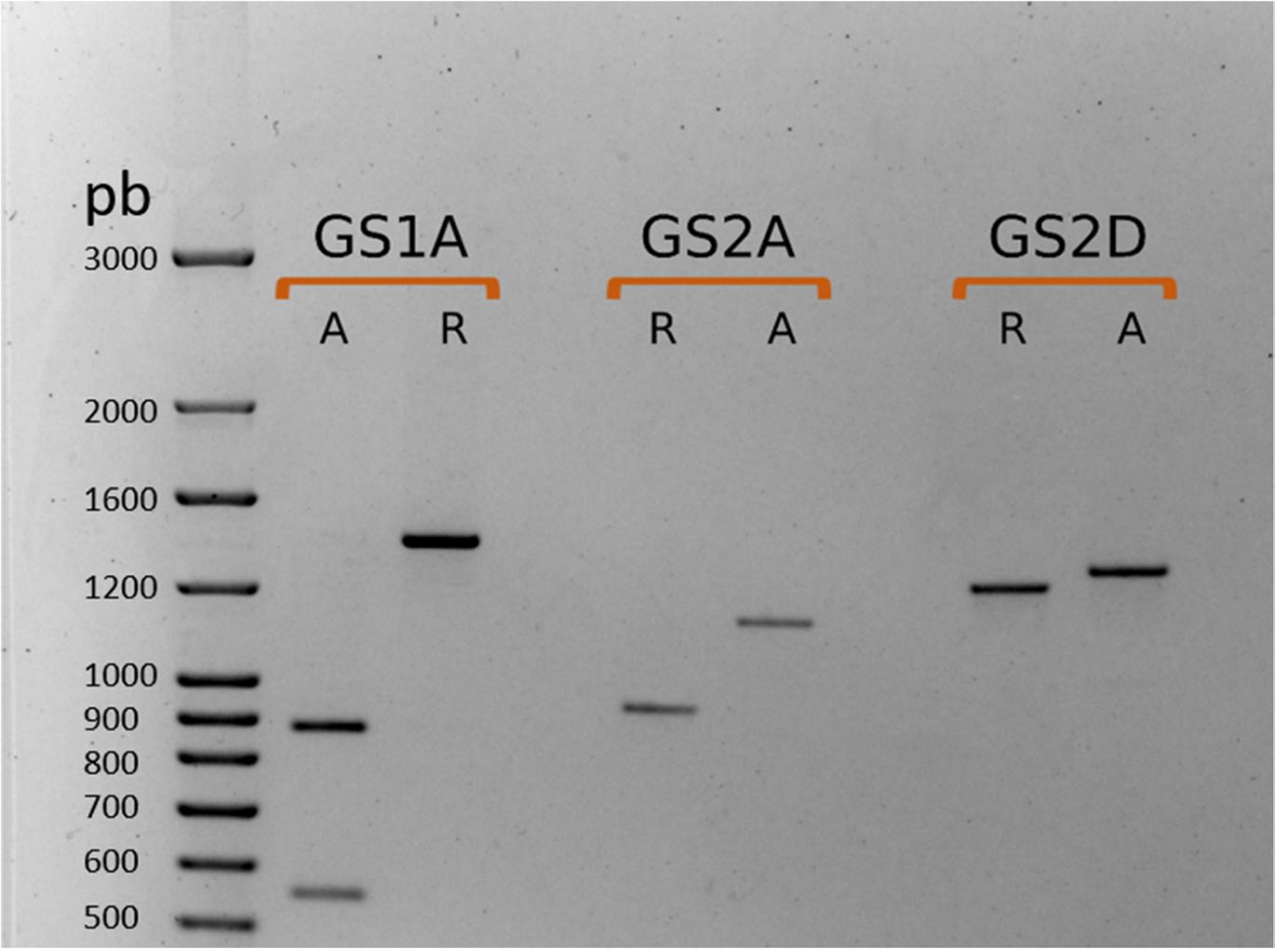
Resolution of alleles of the sequence-based molecular markers developed for *GS1A*, *GS2A* and *GS2D* on agarose gel (1.5%). *GS1A* lanes: R corresponds to the marker allele *LM* (marking the haplotypes *GS1A-hap1* and *-3*) and A to the marker allele *SM* (marking *GS1A-hap2*). *GS2A* lanes: R corresponds to the marker allele *Del* (*GS2A-hap1*) and A to the marker allele *NoDel* (*GS2A-hap2*). *GS2D* lanes: R corresponds to the marker allele *M1* (*GS2D-hap1* and *-hap1bis*) and A to the marker allele *M2* (*GS2D-hap2* and *-hap2bis*). The first lane corresponds to NZYDNA ladder VII (NZYTech, Lisboa)

For *GS1A*, the CAPS marker described above discriminate allelic variants based on the SNP [C/T] present at 1777 from the starting codon. Hence, the restriction enzyme will not cut the amplicon in varieties presenting the *GS1A-hap1* and *GS1A-hap3* (namely *GS1A-LM* marker allele) while the amplicon will be excised in varieties with the *GS1A-hap2* (*GS1A-SM* marker allele). For *GS2A*, the pair of primers designed is able to detect by PCR the presence/absence of the 239 bp deletion. Thus, a band of 935 bp is amplified in varieties with the *GS2A-hap1* sequence (namely *GS2A-Del* marker allele) whereas a band of 1174 bp is obtained for *GS2A-hap2* (*GS2A-NoDel* marker allele). Finally, the 1^st^ amplicon of the *GS2D* gene, containing several INDELs (not shown) was used to discriminate varieties carrying the *GS2D-hap1* against those with the *GS2D-hap2*, a smaller amplicon being obtained in the former (1391pb; namely *GS2D-M1* marker allele) than in the latter (1433pb; *GS2D-M2* marker allele). Figure 2 illustrates the easy resolution of these biallelic markers on agarose gels. The correspondence between molecular marker alleles and haplotypes expected according to their in silico design was further confirmed by marker profiling of the sequenced varieties.

### *GS* marker-trait relationships in the diversity panel

The gene sequence-based markers developed for *GS1A*, *GS2A* and *GS2D* allowed allelic profiling of 99.5, 96.8 and 93.6%, respectively, of the Spanish bread wheat landraces forming the diversity panel (Table 2). Online Resource 10 provides information on the specific marker alleles present in each of the 187 accessions and on their population assignment after the genetic structure analysis conducted on this germplasm collection by Pascual et al. (2020b). *GS1A-SM* was present in almost 85% of varieties. *GS2A-NoDel* was the most frequent allelic alternative at *GS2A* (66% of varieties) while *GS2D-M1* was present in 71% of the genotyped varieties. It can be noted that for *GS1A* and *GS2D* the predominant alleles were the noted above in all the four populations but, contrary to the overall trend, the *GS2A-Del* allele was the most frequent in Pop 4 (Table 2). Two-by-two contingency tests demonstrated independent distribution of the marker alleles for *GS2D* and either *GS1A* and *GS2A* in the diversity panel of varieties. However, some statistical association between *GS1A* and *GS2A* was found, the non-allelic combinations *GS1A-SM*:*GS2A-Del* and *GS1A-LM*:*GS2A-NoDel* being slightly more frequent than expected (Χ^2^ = 4.44, d. f. = 1; *P* = 0.035).

**Table 2:** Frequency of marker variants for genes *GS1A*, *GS2A* and *GS2D* in the 187 bread wheat landraces forming the diversity panel. Their distribution in the 4 populations determined in this germplasm collection in Pascual et al. (2020b) is also indicated

*GS* gene	Marker allele	Pop1	Pop2	Pop3	Pop4	Total N of accessions	%
*GS1A*	*LM*	1	29	-	-	30	16.0
	*SM*	24	80	16	36	156	83.4
	Not determined	-	1	-		1	0.5
*GS2A*	*Del*	10	20	3	29	62	33.2
	*NoDel*	15	86	12	6	119	63.6
	Not determined	-	4	1	1	6	3.2
*GS2D*	*M1*	22	62	14	26	124	66.3
	*M2*	3	38	2	8	51	27.3
	Not determined	-	10	-	2	12	6.4

The results of the statistical analyses conducted to determine the influence of environmental and GS genotype factors, as well as GS × E, on the traits analysed in the diversity panel by López-Fernández et al. (2021) are summarized in Online Resource 11. The effects of GS genes considered in the analysis included not only allelic variation at *GS1A*, *GS2A* and *GS2D*, but also their two-by-two interactions. The environment had always a great effect on the evaluated traits while none of the GS gene × E interaction was significant.

**Fig. 3:**
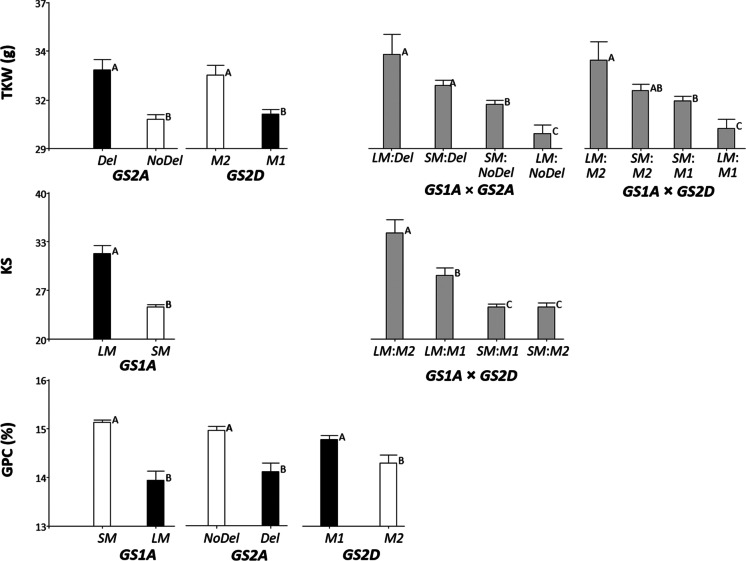
Mean phenotypic value associated to allelic variants and non-allelic combinations for the *GS1A*, *GS2A* and *GS2D* functional markers developed in the study. Only the genes and gene-by-gene interactions with significant effect (*P* < 0.01) on the traits are represented. Among allelic variants, the black bars indicate the Chinese Spring marker allele. Means with a common letter are not statistically different (*P* > 0.05). TKW: thousand-kernel weight; KS: kernels per spike; GPC: grain protein content

TKW was very significantly affected by allelic variation at *GS2* homoeogenes, *GS2A-Del* and *GS2D-M2* having a positive effect on this yield component trait (Fig. 3). *GS1A* did not show to affect TKW when analysed individually, but its interaction with either of those two *GS2* genes was significant (see Online Resource 11). The mean comparison analyses revealed that the favourable alleles for *GS2A* and *GS2D* were both epistatic over *GS1A*; but in their absence (i.e., in genotypes *GS2A-NoDel* or *GS2D-M1*), the *GS1A-SM* allele produced higher TKW than its alternative allele *GS1A-LM* (Fig. 3).

The number of kernels per spike was significantly influenced by *GS1A*, the favourable allele being *GS1A-LM*. The significant interaction between *GS1A* and *GS2D* and mean comparison of the non-allelic combinations for these loci further showed differences for KS between *GS2D* alleles *M1* and *M2* in genotypes carrying the favourable *LM* allele at the *GS1A* locus (Fig. 3; Online Resource 11).

Grain protein content was highly affected by *GS1A* and *GS2A* and also, but to a lower extent, by *GS2D*. The favourable alleles were *GS1A-SM* (as noted above, also beneficial for TKW in certain GS genotype backgrounds), *GS2A-NoDel* (with negative effect on TKW) and *GS2D-M1* (with negative effect on TKW as well as on KS in *GS1A-LM* accessions).

Allelic variation at none of the loci examined showed to influence SVol, a wheat quality parameter tightly associated to gluten strength. No significant interaction among loci was either detected (see Online Resource 11).

## Discussion

### Allelic diversity at *GS1* and *GS2* homoeogenes

Our haplotype characterization in the sequencing panel has revealed a wider allelic variability at the B genome *GS1* and *GS2* genes compared to the A and D genome counterparts (Table 1). Li et al. (2011) characterized the allelic diversity for the *GS2* homoeogenes in the Mini core collection of Chinese wheat, composed of around 150 landraces and 100 commercial varieties, and reported quite similar results, with 2, 6 and 2 haplotypes for *GS2A*, *GS2B* and *GS2D*, respectively. Regarding *GS1* homoeogenes, Guo et al. (2013) found two distinct haplotypes of a gene designated *TaGS1a* (Bernard et al. 2008) in a set of 60 Chinese winter wheat varieties. Guo and coworkers reported mapping of that gene to chromosome 6D, which led them and others to assume that corresponded to the *GS1D* homoeogene (see also Li et al. 2019). However, our alignment of the sequence cloned by Bernard et al. (2008) to the wheat reference genome showed that it corresponds to the *GS1* gene located on chromosome 6A. This has been furtherly confirmed by the successful amplification of the *GS1A* marker reported here (actually based on the same polymorphism used in Guo et al. 2013), in a wide collection of durum wheat landraces (Pascual, unpublished results). To our knowledge, no wide survey of *GS1B* and *GS1D* haplotype variations has yet been documented in common wheat. Nigro et al. (2019) have conducted association mapping of GPC by analysing genotype variation at several NUE-related candidate genes in 240 accessions of tetraploid wheat. However, although *GS1* and *GS2* homoeogenes on the A and B genome were included in the study, data on the allelic diversity found in that germplasm collection are not reported.

Most of the haplotype polymorphisms found in the present study do not provoke protein sequence differences between alleles, the exceptions being a single non-synonymous substitution in *GS2B* and two amino acid substitutions that differentiate the deduced protein sequence of *GS2D* in Chinese Spring and Xiaoyan-54 (Li et al. (2011); Online Resources 3 to 7). This could lead to question that allelic variation at *GS1A*, *GS1B* and *GS2A* may actually be responsible for any of the phenotypic effects reported here. However, non-coding regions (promoters, introns, UTRs) can affect the function of a gene by influencing its RNA processing and/or stability. Actually, a recent study has described differential expression of a glutamine synthetase *GSr* gene, related to differences in TKW, in two wheat varieties which have identical coding regions (Yang et al. 2022).

For the three molecular markers that were genotyped in the diversity panel, we have found that one of the alleles was predominant (Table 2). It makes less unexpected that the Spanish landraces in the sequencing panel shared the same haplotypes for all GS genes but those located on the B genome (Table 1). Li et al. (2011) reported also a quite uneven distribution of allelic alternatives at *GS2* genes, especially at *GS2D*, in the 153 landraces present in the Mini core collection of Chinese wheat varieties. Even at the much more polymorphic *GS2B* locus, these authors found that one out of the 6 existing haplotypes was present in around 70% of the landraces. Their results can be further compared with those reported here since the gene sequences of Chinese Spring have been used as reference in the two studies. Notably, the Chinese Spring *GS2A* allele (namely *GS2A-Del* in the present work) is present in a quite similar proportion in the Chinese and Spanish sets of landraces (33% and 41%, respectively). Regarding *GS2D*, the Chinese Spring allele is the most frequent in both collections but much more abundant in the Chinese local germplasm (95% versus 66% in the present study), its allelic alternative (i.e., the Xiaoyan-54 allele, represented here by *GS2D-M2*) being clearly minority in the two cases.

Khalid et al. (2019) determined the allele frequencies of 87 functional markers in a panel of 213 wheat breeding advanced lines and found a higher frequency of the favourable alleles associated to grain size and weight. In our panel of Spanish landraces, the more frequent alleles of the three genes under analysis were those for which a positive effect on GPC has been detected (Table 2; Fig. 3). This finding could relate with the selection by local farmers of materials with good bread-making properties in a country like Spain, where it is documented that, until the 1930s of the XXth century, most of the wheat production was used for human consumption (Rivero 2013).

### Phenotypic effects of *GS1* and *GS2* genes

Several discrepancies are evidenced between the sequencing and the diversity panel regarding the effects of *GS1A*, *GS2A* and *GS2D* genes on the traits that were evaluated in both germplasm sets: TKW, KS, GPC and SVol. Such discrepancies refer mostly to the finding of a significant effect of a specific GS gene in the diversity panel, but not in the sequencing panel. This holds for *GS1A* on KS and GPC, for GS2A on TKW, and for GS2D on TKW and GPC (Figs. 1 and 3; see also Online Resources 9 and 11). The opposite situation occurs for SVol, where the positive influence of some specific alleles has only been detected in the sequencing panel. Gluten strength of bread wheat varieties is well known to be mainly dependent on two factors: the quantity of protein in grain and the specific high-molecular-weight glutenins encoded by the *Glu-1* homoeogenes *Glu-A1*, *Glu-B1* and *Glu-D1* (e.g., Branlard and Dardevet 1985; Laidig et al. 2022; López-Fernández et al. 2021; Payne et al. 1987). Actually, SVol was included among the parameters under study to determine whether an eventual effect of a given GS gene on GPC could indirectly affect functional quality of wheat flour, which had never been tested before. Our results, however, clearly indicate no relation between both traits associated to variation at GS loci. The significant effects of most GS genes on SVol detected in the sequencing panel must not be really reflecting any functional gene-trait relationship, being likely attributable to the casual combination of specific alleles at GS and *Glu-1* loci in the set of 15 varieties analysed.

The results obtained in the sequencing panel, evaluated by field assays conducted in six environments, can be taken as reliable for that particular group of wheat genotypes, but our study shows that they are not generalizable. To some extent, this might explain the inconsistency among results derived from earlier studies on the influence of GS activity on NUE-related traits in response to external traits — necessarily based on a single or a few genotypes — especially since between-genotype differences for the level and pattern of GS expression have been reported (Bernard et al. 2008; Gayatri et al. 2021; Thomsen et al. 2014). All the discussed below is then based on our results from the diversity panel mostly contrasted with studies conducted on large number of genotypes, either wide germplasm panels or biparental populations.

To facilitate further discussion, a summary of the main findings of the marker-trait association study conducted on the diversity panel is presented in Table 3. It must be noted that the findings reported in López-Fernández et al. (2021) on the effect of the genetic structure of the Spanish collection of bread wheat landraces on TKW, KS, GP and SVol support that no relationship exists between the differences among populations at *GS2A* noted above (Table 2) and the phenotypic variation in this germplasm panel.

**Table 3:** Summary of alleles showing beneficial effects on thousand-kernel weight, number of kernels per spike and grain protein content. The marker alleles corresponding to the Chinese Spring *GS1A*, *GS2A* and *GS2D* gene sequences are, respectively, *LM*, *Del* and *M1*. The *GS2D-M2* allele corresponds to the Xiaoyan-54 haplotype. Varieties presenting the *GS1A* and *GS2A* sequences marked by the *SM* and *NoDel* alleles (*GS1A-hap2* and *GS2A-hap2*, respectively) can be found in Table 1

	*GS1A*	*GS2A*	*GS2D*
TKW	SM* (*NoDel*, *M1*)	*Del*	*M2*
KS	*LM*		M2* (*LM*)
GPC	*SM*	*NoDel*	*M1*

*TKW*, thousand-kernel weight; *KS*, kernels per spike; *GPC*, grain protein content

^*^ Allele whose positive effect is only detected in genotypes that present the non-allelic variant(s) noted in brackets

The spatial–temporal changes in the relative activity of GS1 and GS2 isozymes during plant development have led to suggest that the cytosolic GS1 isoform is mainly involved in grain traits determined at post-anthesis (Bernard et al. 2008; Zhang et al. 2017c). However, its central role in the remobilization of N to the developing spike, when the number of fertile florets is being stablished, has also been suggested (Thomsen et al. 2014). Confirming this view, we have demonstrated that genotype variation at the *GS1A* locus may influence KS, a critical component of yield potential determined at the pre-anthesis stage. To our knowledge, no earlier report has documented the positive effect of a wheat *GS1* allele on this NUE-related trait. Our study has further found that opposite *GS1A* allelic variants have a positive influence on the pre- and post-anthesis traits analysed; i.e., *GS1A-LM* on KS and *GS1A-SM* on GPC (Fig. 3). This finding may provide one piece on the puzzling genetic network underlying the general observation that the number of grains per ear is negatively related to size and protein content of grains (Habash et al. 2007; reviewed in Teng et al. 2022).

By conducting marker to trait association analyses, Guo et al. (2013) and Li et al. (2019) have reported some discrepant results on the effect of the Chinese Spring *GS1A* allele on TKW. So, while the first authors detected a positive influence of this allele in a RIL population, Li and coworkers have not found any significant difference between allelic variants at *GS1A* in a wide collection of winter wheat lines. Our results are partly in agreement with the latter study since no significant influence of *GS1A* on TKW was detected when its individual effect was tested in our set of landraces (Online Resource 11). However, our gene-by-gene interaction analysis has shown that this locus behaves as hypostatic of either *GS2A-Del* and *GS2D-M2* marker alleles (Fig. 3). But contrarily to the reported by Guo and coworkers, the Chinese Spring allele (*GS1A-LM*) was the associated to lower TKW when some effect of this locus was detected in our study. The discovery of genetic factors which simultaneously increase TKW and GPC is a matter of great breeding interest in order to select genotypes where the usual inverse relation between both traits can be broken (Cormier et al. 2016). According to the results reported here, the *GS1A-SM* allele could be a good candidate for that aim.

It can be added that Habash et al. (2007) colocalized large-effect QTLs for GPC and TKW with the *GS1* locus on 6B (namely, *GS1B*) as well as a major QTL for KS on 6DL, where *GS1D* is located. However, none of these *GS1* homoeogenes could be included in our marker-trait study for having been found to be too polymorphic (*GS1B*) or monomorphic (*GS1D*) among the accessions in the sequencing panel.

Regarding the wheat homoeogenes coding for the plastidic GS isoform, our results show significant effects of both *GS2A* and *GS2D* on TKW and GPC in the diversity panel of landraces (Fig. 3; Table 3). By studying a RIL population, Li et al. (2011) detected that *GS2A* influenced TKW and GPC when genotypes were cultivated under low-N conditions, the increasing effect being attributed to the Xiaoyan-54 *GS2A* allele. Overexpression of this allele by Hu et al. (2018) demonstrated later its beneficial influence on GPC at any N regime; but its improving effect on TKW was not consistent in all transgenic lines and treatments. The *GS2A-hap2* allele found in 12 out of 15 varieties of the sequencing panel is almost identical to the Xiaoyan-54 allele, presenting a unique additional SNP at position 1722 from the start codon when both are compared with the Chinese Spring *GS2A* sequence (see Online Resource 5 for comparison between *GS2A-hap2* and the Chinese Spring allele). It is then likely that they are functionally similar, which closely agrees with the finding of higher GPC in landraces presenting the *GS2A-NoDel* marker allele at *GS2A* locus. For TKW our study indicates some increasing effect of the Chinese Spring allele (*GS2A-Del* marker allele). This is not coincident with the reported in Li et al. (2011) but it is less unexpected according to the inconsistent results on TKW obtained after the transgenic overexpression of the Xiaoyan-54 *GS2A* sequence (Hu et al. 2018). The QTL mapping approach Habash et al. (2007) detected higher GS2 enzymatic activity in genotypes bearing this allele, but no impact of *GS2A* genotype on the traits evaluated (which included KS, TKW and GPC) was evidenced. These authors neither found colocalization between any QTL for GS activity and some large-effect QTLs for TKW and GPC that could be associated to *GS2B*. All that reinforces the doubtful reliability of using GS activity to predict improved NUE phenotypes as has been suggested in some instances (Cormier et al. 2016; Zhang et al. 2017c).

Li et al. (2011) studied the effect of *GS2D* in a doubled haploid population that segregated for the Chinese Spring and Xiaoyan-54 alleles, the same allelic variants contrasted in our study (namely, *GS2D-M1* and *GS2D-M2*). These authors found that the Chinese Spring allele was associated with higher TKW, and also higher N uptake before flowering. On the contrary, our results have indicated that genotypes bearing the Xiaoyan-54 allele have higher TKW, but mean comparison analysis of non-allelic combinations at *GS1A* and *GS2D* further reveals that the improving effect of *GS2D-M2* is not significant in landraces bearing the *GS1A-SM* allele (Fig. 3). Regarding GPC, we have detected a positive effect of the Chinese Spring *GS2D-M1*, which also disagrees the lack of any influence of *GS2D* on grain N content (equivalent to GPC) reported by (Li et al. 2011). Nevertheless, and although the interaction between *GS2A* and *GS2D* was not significant at the threshold of 0.01 (*P* = 0.0187; see Online Resource 11), mean comparison between non-allelic combinations at these loci suggests that such effect on GPC could be masked in genotypes bearing the beneficial GS2A-*NoDel* allele at *GS2A* locus.

*GS2D* has also showed to affect KS but only in genotypes where the beneficial *GS1A-LM* allele is present. Considering all cases of genetic interactions reported in the present study, this is the unique instance where pyramiding of favourable alleles would be clearly successful to improve a NUE-related trait (Table 3; Fig. 3). It can be noted that, as occurred for *GS1A*, the two *GS2D* allelic variants seem to exert opposite effects on the improvement of KS and GPC.

### Overview

In spite that physiological connections must exist between the enzymatic activities of cytosolic and plastidic glutamine synthetase, no earlier report has documented the influence of combined allelic variation at *GS1* and *GS2* homoeogenes on NUE-related traits in wheat. Nigro et al. (2019) included the A and B homoeogenes coding for both glutamine synthetase families in their association study of GPC in durum wheat. However, their effects were analysed on a gene-by-gene basis, providing no clue on genetic interactions that could eventually explain part of the trait variation characterized in the panel of genotypes under study. The same holds for studies that have been focused on the effect of individual homoeogenes of a given GS family (i.e., Li et al. 2011).

Overall, the present study has revealed that epistatic interactions can mask the effect of individual loci or alleles. Between-gene interactions are acknowledged as a component of phenotypic variation of complex polygenic traits in text-books and theoretical papers. However, they are commonly ignored in marker-trait association studies attempting to identify alleles that can predict a favourable phenotype for NUE-related traits. In agreement with the reported here, Li et al. (2019) have demonstrated that one allele of a given locus, earlier classified as having a positive effect on TKW and kernel associated traits, can be not so beneficial when combined with particular alleles at other loci. That study further showed that the Chinese Spring *GS1A* allele, for which a positive effect on TKW had been demonstrated (Guo et al. 2013) was not present in the allele combinations that presented the genotypes with the highest TKW values. It is remarkable that, as noted above, our results indicate not only that the *GS1A* locus behaves as hypostatic of *GS2A* and *GS2D*, but also that the Chinese Spring allele (*GS1A-LM* in the present study) is associated to lower TKW (Table 3).

The knowledge of the epistatic effects that may exist between target genes can be critical for the success of breeding strategies aiming to pyramid favourable alleles. Additionally, genetic interaction effects may provoke inconsistencies and discrepancies between gene/QTL mapping studies of complex polygenic traits, like NUE components, especially if conducted on materials with narrow genetic segregation (i.e., DH, RILs and, especially, NILs populations). Its allopolyploid nature makes the handling of epistatic effects a much more troublesome challenge in wheat than in other major crops. Because of their practical implications, validation of the marker-trait relationships detected here on other wheat germplasm collections would be of great interest.

## Supplementary Information

Below is the link to the electronic supplementary material.

Supplementary file1 (PDF 83 KB)

Supplementary file2 (PDF 85 KB)

Supplementary file3 (XLSX 14.4 KB)

Supplementary file4 (XLSX 12 KB)

Supplementary file5 (XLSX 11.6 KB)

Supplementary file6 (XLSX 13.9 KB)

Supplementary file7 (XLSX 11.4 KB)

Supplementary file8 (PDF 162 KB)

Supplementary file9 (PDF 66.9 KB)

Supplementary file10 (PDF 178 KB)

Supplementary file11 (PDF 63.8 KB)

## Data Availability

Raw data from field experiments of varieties forming the GS sequencing panel are provided as supplementary material (Online Resource 8). Raw data from field experiments of varieties forming the diversity panel of Spanish landraces are provided as supplementary material in López-Fernández et al. (2021), while their molecular marker profiling is provided in Online Resource 10.

## Material availability

All landraces used in the study are distributed under request by the Spanish germplasm bank of genetic resources (Centro de Recursos Fitogenéticos) at Instituto Nacional de Investigación y Tecnología Agraria y Alimentaria (INIA), CSIC, Alcalá de Henares, Madrid, Spain.
