# Supplementary material for: Development and marker-trait relationships of functional markers for glutamine synthetase GS1 and GS2 homoeogenes in bread wheat
Source: Mol Breed. 2023 Jan 19;43(2):8. doi: 10.1007/s11032-022-01354-0 (PMC10248667; doi:10.1007/s11032-022-01354-0)
Supplement: Supplementary file 1 — Supplementary file1 (PDF 83 KB) [file 11032_2022_1354_MOESM1_ESM.pdf]

Title: Development and marker-trait relationships of functional markers for glutamine synthetase *GS1* and *GS2* homoeogenes in bread wheat
Journal: Molecular Breeding
Authors: Pascual L. Solé-Medina A. Faci I. Giraldo P. Ruiz M and Benavente E.
Corresponding author: E. Benavente; Department of Biotechnology-Plant Biology. Universidad Politécnica de Madrid. Madrid. Spain; e.benavente@upm.es

Online Resource 1. Primers combinations and melting temperatures employed to amplify the *GS1* and *GS2* homoeogenes in the varieties of the sequencing panel.

Gene	Amplicon	TM (°C)	Forward primer	Reverse primer	Size pb
GS1A	1st	60	TGGGTCGTCGCCATTATATAGCC	CTCGTTAGTCCGTCGTAGTTGAG	1683
GS1A	2nd	60	GCGAGGTCATCCTGTAGTAAGTG	CTCCCACTGAAAACCAAACATAG	1405
GS1A	3th	60	TACAAGGCCTGCCTCTTTGC	ATTGATGATTACACGAGGACCAG	1242
GS1B	1st	62.5	TGGGTCGTCGCCATTATATAGCC	CTGTCGTAGTTGACCAGAACTCC	1675
GS1B	2nd	60	GCGAGGTCATCCTGTAGTAAGTG	ATCACAAAAGCCTCCTCCCTTATC	1489
GS1B	3th	57	TACAAGGCCTGCCTCTTTGC	TTGATGATTACACGAGACGAGAC	1234
GS1D	1st	57	TGGGTCGTCGCCATTATATAGCC	TCTTAATGGAACTCGTTGCTCTC	1660
GS1D	2nd	60	GCGAGGTCATCCTGTAGTAAGTG	GAAAGGGAGAGCACCATTTAATC	1423
GS1D	3th	60	TACAAGGCCTGCCTCTTTGC	AATACCATCCGTCCATCCGTAG	1326
GS2A	1st	60	AGAGCCCTATCGCCTAATCAAGAG	TTAGCATAAAGCACGTCCAGATGA	1620
GS2A	2nd	60	GTCAGAGCTACCCAAATGGAATTAT	ATATACCTGGATTGGTTTTGGGTCA	1450
GS2A	3th	59	TCATCCTTTTGCTGTTATCAATTTT	AATAACGTAGTTCTCCGAATGGAC	1533
GS2B	1st	60	AGAGCCCTATCGCCTAATCAAGAG	GGCTGAAAGCAGGATAGAAAAACA	1528
GS2B	2nd	59	ATTACTGTCCATGTTTTCGGCTTTT	ATATACCTGGATTGGTTTTGGGTCA	1384
GS2B	3th	60	GCCCTTCGTAAATACCATAAAAACAG	AATAACGTAGTTCTCCGAATGGAC	1555
GS2D	1st	54	CGAGATGTGTGTGCATTCTGG	TGTTGTTGCCTCCTCGGAAT	1659
GS2D	2nd	55	GCACATGCAGAGTAATCTGGATTTC	ATATACCTGGATTGGTTTTGGGTCA	1280
GS2D	3th	58	AACATCAGATCCCATAAGTCCATGA	AATAACGTAGTTCTCCGAATGGAC	1708
