# Supplementary material for: Development and marker-trait relationships of functional markers for glutamine synthetase GS1 and GS2 homoeogenes in bread wheat
Source: Mol Breed. 2023 Jan 19;43(2):8. doi: 10.1007/s11032-022-01354-0 (PMC10248667; doi:10.1007/s11032-022-01354-0)
Supplement: Supplementary file 2 — Supplementary file2 (PDF 85 KB) [file 11032_2022_1354_MOESM2_ESM.pdf]

Title: Development and marker-trait relationships of functional markers for glutamine synthetase *GS1* and *GS2* homoeogenes in bread wheat
Journal: Molecular Breeding
Authors: Pascual L. Solé-Medina A. Faci I. Giraldo P. Ruiz M and Benavente E.
Corresponding author: E. Benavente; Department of Biotechnology-Plant Biology. Universidad Politécnica de Madrid. Madrid. Spain; e.benavente@upm.es

Online Resource 2. Details of the field experimental conditions in the six environments where the 15 varieties forming the sequencing panel were evaluated. The agronomic and quality-related traits analyzed in each trial are indicated.

Trial code	Site	Locality	Year (harvest)	Plot size (m^2)	Replications	GY	TW	TKW	SN	KS	GPC	SVol
LC15	La Canaleja	Alcalá de Henares	2015	12	3	yes	yes	yes	yes	yes	yes	yes
LC16	La Canaleja	Alcalá de Henares	2016	3,6	3	no	yes	yes	yes	yes	yes	yes
LC17	La Canaleja	Alcalá de Henares	2017	12	2	yes	yes	yes	yes	yes	yes	yes
LG16	Limagrain Station	Elorz	2016	12	2	yes	yes	yes	no	no	yes	yes
LG18	Limarain Station	Elorz	2018	12	2	yes	yes	yes	no	no	yes	yes
UPM17	UPM	Madrid	2017	1	1	no	no	yes	no	yes	yes	yes

GY: Grain yield; TW: test weight; TKW: thousand-kernel weight; SN: spike number per square metre; KS: kernels per spike; GPC: grain protein content; SVol: SDS-sedimentation volume.
