# Supplementary material for: Development and marker-trait relationships of functional markers for glutamine synthetase GS1 and GS2 homoeogenes in bread wheat
Source: Mol Breed. 2023 Jan 19;43(2):8. doi: 10.1007/s11032-022-01354-0 (PMC10248667; doi:10.1007/s11032-022-01354-0)
Supplement: Supplementary file 8 — Supplementary file8 (PDF 162 KB) [file 11032_2022_1354_MOESM8_ESM.pdf]

Title: Development and marker-trait relationships of functional markers for glutamine synthetase GS1 and GS2 homoeogenes in bread wheat

Journal: Molecular Breeding

Authors: Pascual L, Solé-Medina A, Faci I, Giraldo P, Ruiz M, Benavente E.

Corresponding author: E. Benavente; Department of Biotechnology-Plant Biology, Universidad Politécnica de Madrid, Madrid, Spain; e.benavente@upm.es

Online Resource 8. Data set for statistical analyses of the traits evaluated in field trials conducted with the bread wheat varieties of the sequencing panel.

Mean value of replications (3 in LC15 and LC16 trials; 2 in LC17, LG16 and LG18 trials) is indicated.

GY: Grain yield; TW: test weight; TKW: thousand-kernel weight; SN: spike number per square metre; KS: kernels per spike; GPC: grain protein content; SVol: SDS-sedimentation volume.

Trial	Variety	GY (kg/ha, 12% humidity)	TW (kg/hL)	TKW (g, 12% humidity)	SN (m^{-2})	KS	GPC (%, d.m.b.)	Svol (mm)
LC15	NOGAL	3683	70,6	23,2	511	31,2	15,4	100,5
LC15	ARTUR NICK	4798	72,1	26,5	578	31,8	12,0	76,0
LC15	CALIFA SUR	4201	69,6	24,2	644	27,6	15,1	90,7
LC15	BERDUN	2597	69,5	22,9	482	23,5	15,1	89,5
LC15	GAZUL	3846	74,6	30,2	447	28,9	15,0	100,2
LC15	ABLACA	4530	64,2	21,5	633	27,1	16,3	103,8
LC15	MARIUS	4042	66,0	32,4	607	18,8	15,1	67,7
LC15	ANZA	4825	71,9	28,2	518	34,4	13,8	73,7
LC15	YECORA	5200	71,8	28,9	551	33,6	14,7	111,8
LC15	PANE-247	4358	69,9	28,9	456	32,6	15,7	60,7
LC15	ARAGON-03	4371	75,3	30,8	729	19,7	15,1	65,0
LC15	CHAMORRO	3232	70,2	32,5	544	18,5	15,0	99,2
LC15	MOCHO ROJO	2677	71,7	32,7	709	11,6	16,7	48,2
LC15	CANDEAL VELLISCA	3121	74,7	30,3	762	13,9	17,7	101,5
LC15	ROJO CARAVACA	3794	72,4	26,0	793	18,8	14,5	65,3
LC16	NOGAL		72,8	28,5	703	30,9	14,4	71,7
LC16	ARTUR NICK		77,6	37,6	547	44,4	12,7	65,0
LC16	CALIFA SUR		74,9	33,7	740	22,8	12,3	72,3
LC16	BERDUN		72,3	28,3	618	43,3	13,5	79,2
LC16	GAZUL		76,6	36,8	607	41,6	14,7	91,5
LC16	ABLACA		67,1	32,6	651	38,2	16,0	100,2
LC16	MARIUS		71,1	36,7	731	23,5	13,9	63,0
LC16	ANZA		79,6	34,1	724	21,9	14,0	60,5
LC16	YECORA		75,2	39,4	590	28,8	15,6	99,8
LC16	PANE-247		75,0	36,8	569	33,5	13,9	58,3
LC16	ARAGON-03		75,4	37,8	707		16,4	55,5
LC16	CHAMORRO		71,6	45,3	542	17,5	18,4	112,5
LC16	MOCHO ROJO		71,1	39,8	704	18,3	17,2	41,5
LC16	CANDEAL VELLISCA		76,5	40,9	727	20,5	17,5	107,5
LC16	ROJO CARAVACA		74,2	36,2	950	24,7	16,7	30,8

Trial	Variety	GY (kg/ha, 12% humidity)	TW (kg/hL)	TKW (g, 12% humidity)	SN (m^{-2})	KS	GPC (%, d.m.b.)	Svol (mm)
LC17	NOGAL	4835	73,9	27,5	510	34,9	16,8	86,8
LC17	ARTUR NICK	3999	75,9	29,4	387	34,7	15,3	84,5
LC17	CALIFA SUR	4590	74,4	28,7	387	41,8	15,0	88,3
LC17	BERDUN	5708	74,1	26,3	557	38,6	14,9	96,3
LC17	GAZUL	3090	76,9	34,3	290	27,2	17,6	92,5
LC17	ABLACA	2869	70,0	28,7	303	31,6	18,4	101,3
LC17	MARIUS	2081	70,2	33,5	260	24,8	13,9	65,8
LC17	ANZA	3562	78,3	33,3	363	31,2	15,2	61,8
LC17	YECORA	4651	75,6	35,3	443	30,9	14,4	99,0
LC17	PANE-247	3480	73,3	32,4	363	29,7	19,7	53,5
LC17	ARAGON-03	3460	76,0	33,6	587	16,7	18,2	50,3
LC17	CHAMORRO	2280	71,5	36,4	437	14,6	18,3	88,0
LC17	MOCHO ROJO	2300	74,1	36,0	427	14,8	18,5	52,3
LC17	CANDEAL VELLISCA	2078	75,3	35,1	383	15,2	19,1	84,5
LC17	ROJO CARAVACA	2005	76,4	31,3	397	16,9	18,0	36,0
LG16	NOGAL	7363	64,3	30,4			14,2	81,0
LG16	ARTUR NICK	7527	63,7	38,6			14,1	64,0
LG16	CALIFA SUR							
LG16	BERDUN	7802	68,2	31,9			14,4	74,5
LG16	GAZUL	8077	71,2	43,1			16,0	97,0
LG16	ABLACA	7637	68,8	39,5			15,5	104,0
LG16	MARIUS	7802	60,8	43,3			13,0	64,0
LG16	ANZA	5604	54,6	27,6			14,3	52,5
LG16	YECORA	4670	55,5	40,5			15,7	105,0
LG16	PANE-247	3901	60,2	38,0			16,3	46,5
LG16	ARAGON-03	3297	60,7	36,6			16,5	47,5
LG16	CHAMORRO	2143	54,1	37,8			15,3	92,0
LG16	MOCHO ROJO	2363	58,1	39,1			17,0	40,0
LG16	CANDEAL VELLISCA	3571	58,1	40,1			17,1	102,0
LG16	ROJO CARAVACA	2582	62,8	35,9			15,5	36,5
LG18	NOGAL	9121	69,7	35,7			12,8	84,0
LG18	ARTUR NICK	9835	69,4	32,7			13,0	78,5
LG18	CALIFA SUR	9615	70,0	37,7			11,9	79,5
LG18	BERDUN	9396	69,4	38,8			12,8	79,5
LG18	GAZUL	8516	70,6	37,7			13,3	93,0
LG18	ABLACA	7033	68,6	33,7			15,0	102,5
LG18	MARIUS	8571	69,3	45,3			13,3	61,5
LG18	ANZA	7637	69,6	30,6			12,9	55,0
LG18	YECORA	7967	69,3	38,6			13,7	95,5
LG18	PANE-247	5879	69,9	47,1			12,9	51,5
LG18	ARAGON-03	4725	69,6	41,8			15,5	32,5
LG18	CHAMORRO	4396	69,1	43,1			14,9	74,0
LG18	MOCHO ROJO	4176	68,1	40,8			17,4	64,0

Trial	Variety	GY (kg/ha, 12% humidity)	TW (kg/hL)	TKW (g, 12% humidity)	SN (m^{-2})	KS	GPC (%, d.m.b.)	Svol (mm)
LG18	CANDEAL VELLISCA	3736	68,1	41,9			16,4	72,5
LG18	ROJO CARAVACA	5000	69,8	40,2			15,5	35,5
UPM-17	NOGAL			24,7		62,0	13,6	79,5
UPM-17	ARTUR NICK			26,0		73,2	16,8	77,0
UPM-17	CALIFA SUR			36,6		57,3	14,1	86,0
UPM-17	BERDUN			21,8		62,3	15,5	86,0
UPM-17	GAZUL			32,3		63,1	16,9	80,0
UPM-17	ABLACA			29,1		66,1	16,4	91,0
UPM-17	MARIUS			38,8		42,5	13,9	56,5
UPM-17	ANZA			36,0		61,0	13,8	57,5
UPM-17	YECORA			33,6		59,4	16,0	97,5
UPM-17	PANE-247			23,1		41,1	17,2	47,0
UPM-17	ARAGON-03			34,7		35,8	16,2	54,0
UPM-17	CHAMORRO			38,1		27,3	16,4	110,5
UPM-17	MOCHO ROJO			30,2		21,3	18,4	41,5
UPM-17	CANDEAL VELLISCA			41,4		24,1	16,0	113,0
UPM-17	ROJO CARAVACA			24,3		33,3	17,5	42,5
