# Supplementary material for: Development and marker-trait relationships of functional markers for glutamine synthetase GS1 and GS2 homoeogenes in bread wheat
Source: Mol Breed. 2023 Jan 19;43(2):8. doi: 10.1007/s11032-022-01354-0 (PMC10248667; doi:10.1007/s11032-022-01354-0)
Supplement: Supplementary file 9 — Supplementary file9 (PDF 66.9 KB) [file 11032_2022_1354_MOESM9_ESM.pdf]

Title: Development and marker-trait relationships of functional markers for glutamine synthetase *GS1* and *GS2* homoeogenes in bread wheat
Journal: Molecular Breeding
Authors: Pascual L, Solé-Medina A, Faci I, Giraldo P, Ruiz M and Benavente E.
Corresponding author: E. Benavente; Department of Biotechnology-Plant Biology, Universidad Politécnica de Madrid, Madrid, Spain; e.benavente@upm.es

Online Resource 9. P values of the F statistics from the analyses of variance of traits evaluated in the 15 wheat varieties of the sequencing panel considering the distinct GS genes and environment (E) as sources of variation. None of the GS × E interactions was significant.

Source of variation	GY	TW	TKW	SN	KS	GPC	SVol
GS1A	0.024	ns	ns	0.0275	0.0289	ns	0.0001
E	<0.0001	<0.0001	<0.0001	<0.0001	<0.0001	0.0010	ns
GS1B	ns	ns	ns	ns	ns	ns (1)	<0.0001
E	<0.0001	<0.0001	<0.0001	<0.0001	<0.0001	0.0009	ns
GS2A	ns (1)	ns	ns	ns	0.0118	0.0062	<0.0001
E	<0.0001	<0.0001	<0.0001	<0.0001	<0.0001	0.0102	ns
GS2B	ns	ns (1)	0.0025	ns	ns	ns	ns
E	0.0021	<0.0001	0.0002	0.0020	0.0027	0.0119	ns
GS2D	ns	ns	ns	0.0308	ns	ns	ns
E	<0.0001	<0.0001	<0.0001	<0.0001	0.0009	0.0017	ns

GY: Grain yield; TW: test weight; TKW: thousand-kernel weight; SN: spike number per square metre; KS: kernels per spike; GPC: grain protein content; SVol: SDS-sedimentation volume.

ns= $P > 0.05$; (1) Means in varieties with different haplotypes are significantly different at $P = 0.05$.
