# Supplementary material for: Development and marker-trait relationships of functional markers for glutamine synthetase GS1 and GS2 homoeogenes in bread wheat
Source: Mol Breed. 2023 Jan 19;43(2):8. doi: 10.1007/s11032-022-01354-0 (PMC10248667; doi:10.1007/s11032-022-01354-0)
Supplement: Supplementary file 10 — Supplementary file10 (PDF 178 KB) [file 11032_2022_1354_MOESM10_ESM.pdf]

Title: Development and marker-trait relationships of functional markers for glutamine synthetase GS1 and GS2 homoeogenes in bread wheat
Journal: Molecular Breeding
Authors: Pascual L, Solé-Medina A, Faci I, Giraldo P, Ruiz M, Benavente E.
Corresponding author: E. Benavente; Department of Biotechnology-Plant Biology, Universidad Politécnica de Madrid, Madrid, Spain; e.benavente@upm.es

Online Resource 10. Allelic variants for the *GS1A, GS2A and GS2D* markers in the 187 bread wheat landraces forming the diversity panel.
The germaplasm bank code (BGE) of each accession and their population assignment (Pop) according to Pascual et al. (2020b) is indicated.

BGE	Pop	**GS1A**	**GS2A**	**GS2D**
BGE000084	4	SM	Del	M2
BGE001942	2	LM	NoDel	M1
BGE001945	3	SM	NoDel	M1
BGE001981	4	SM	Del	M1
BGE003546	4	SM	Del	M1
BGE003614	4	SM	Del	M2
BGE005443	2	LM	NoDel	M1
BGE008209	4	SM	Del	M2
BGE008211	2	SM	NoDel	M2
BGE010003	3	SM	NoDel	M2
BGE011872	4	SM	Del	M2
BGE011886	2	SM	NoDel	M2
BGE011930	2	SM	NoDel	M2
BGE011932	2	SM	NoDel	M2
BGE011940	4	SM	NoDel	M1
BGE011950	2	LM	NoDel	M2
BGE011985	2	LM	NoDel	M1
BGE011995	1	SM	NoDel	M1
BGE012001	2	SM	NoDel	M1
BGE012011	1	SM	NoDel	M1
BGE012034	1	SM	NoDel	M1
BGE012063	1	SM	Del	M1
BGE012068	1	SM	NoDel	M2
BGE012111	2	SM	NoDel	M1
BGE012129	1	SM	NoDel	M1
BGE012132	2	SM	NoDel	M1
BGE012185	2	LM	Del	M1
BGE012192	1	SM	NoDel	M1
BGE012239	2	SM	NoDel	M1

BGE	Pop	GS1A	GS2A	GS2D
BGE012292	2	SM	NoDel	M1
BGE012752	2	SM	NoDel	M2
BGE012860	2	SM	Del	M2
BGE012867	2	SM	Del	M1
BGE012870	3	SM	NoDel	M1
BGE012881	2	SM	Del	M1
BGE012886	2	SM	NoDel	M1
BGE012889	2	SM	Del	M2
BGE012895	2	SM	Del	M2
BGE013128	2	LM	NoDel	M1
BGE013137	2	SM	Del	M1
BGE013155	2	SM	NoDel	M1
BGE013157	2	SM	NoDel	M1
BGE013173	2	LM	NoDel	M1
BGE013174	2	SM	NoDel	M2
BGE013182	2	SM	NoDel	M1
BGE013187	4	SM	Del	M2
BGE013202	3	SM	Del	M1
BGE013203	3	SM	NoDel	M1
BGE013206	3	SM	Del	M1
BGE013211	2	LM	NoDel	M2
BGE013760	4	SM	Del	M1
BGE013777	2	LM	NoDel	M1
BGE013780	2	SM	Del	M2
BGE013801	2	SM	Del	M1
BGE014290	2	SM	NoDel	M2
BGE015376	1	SM	Del	M1
BGE015378	1	SM	NoDel	M1
BGE015380	1	SM	Del	M1
BGE015384	2	SM	NoDel	M1
BGE015399	2	LM	NoDel	M2
BGE015402	4	SM	Del	M1
BGE018217	2	LM	NoDel	M1
BGE018221	2	SM	NoDel	M1
BGE018225	2	SM	NoDel	M2
BGE018232	3	SM	Del	M1
BGE018253	2	SM	NoDel	
BGE018356	2	LM	NoDel	M2
BGE018671	2	SM	NoDel	M1
BGE018922	1	SM	Del	M1
BGE018927	2	SM	Del	M1
BGE019328	1	SM	Del	M1
BGE020362	2	SM	NoDel	M1
BGE020365	2	SM	NoDel	M1

BGE	Pop	GS1A	GS2A	GS2D
BGE020366	2	SM	NoDel	M2
BGE022235	1	SM	NoDel	M1
BGE023723	2	LM	NoDel	M1
BGE023725	4	SM	NoDel	M1
BGE024863	4	SM	NoDel	M1
BGE024866	2	SM	NoDel	M2
BGE024870	2	LM	NoDel	M2
BGE026951	2	SM	NoDel	M2
BGE029105	4	SM	NoDel	M1
BGE029795	4	SM	Del	M1
BGE034186	2	LM	Del	M1
BGE001944	2	SM	NoDel	M2
BGE001983	2	SM	NoDel	M1
BGE002012	2	LM	NoDel	M1
BGE002013	2	SM	NoDel	M1
BGE003156	4	SM	Del	M1
BGE003236	4	SM	Del	M1
BGE003611	4	SM	Del	M2
BGE003612	2	LM	NoDel	M1
BGE003615	4	SM	Del	M1
BGE004791	4	SM	Del	M1
BGE008205	2	SM	NoDel	M1
BGE008220	3	SM	NoDel	M1
BGE008221	2	SM	NoDel	M1
BGE008224	2	LM	Del	M1
BGE008229	2	SM	NoDel	M1
BGE011827	2	SM	Del	M2
BGE011829	2	SM	NoDel	M2
BGE011866	2	LM	NoDel	M2
BGE011869	2	SM	NoDel	M2
BGE011871	2	SM	NoDel	M1
BGE011879	4	SM	NoDel	M1
BGE011882	4	SM	Del	M1
BGE011887	2	SM	NoDel	M2
BGE011888	2	SM	NoDel	M1
BGE011931	1	SM	NoDel	M2
BGE012036	4	SM	NoDel	M1
BGE012045	1	SM	NoDel	M1
BGE012047	1	SM	NoDel	M1
BGE012067	1	SM	Del	M1
BGE012078	2	SM	Del	M2
BGE012120	2	SM	NoDel	M2
BGE012121	2	SM	NoDel	M2
BGE012191	2	SM	Del	M1

BGE	Pop	GS1A	GS2A	GS2D
BGE012194	1	SM	NoDel	M1
BGE012196	4	SM	Del	M1
BGE012198	1	SM	Del	M1
BGE012201	2	SM	NoDel	M2
BGE012205	2	SM	NoDel	M1
BGE012208	2	SM	NoDel	M2
BGE012210	2	SM	NoDel	M2
BGE012213	4	SM	Del	M2
BGE012238	2	SM	NoDel	M2
BGE012578	2	LM	NoDel	M1
BGE012601	2	SM	Del	M1
BGE012603	2	SM	NoDel	M1
BGE012615	2	SM	NoDel	M2
BGE012741	2	LM	NoDel	M2
BGE012805	2	SM	NoDel	M1
BGE012887	2			M1
BGE012890	2	SM		M2
BGE012893	3	SM	NoDel	M1
BGE012894	4	SM	Del	M1
BGE012896	2	LM	NoDel	M1
BGE013149	2	SM	NoDel	
BGE013152	2	SM	NoDel	
BGE013156	2	LM	NoDel	
BGE013179	4	SM	Del	
BGE013183	2	SM	NoDel	M1
BGE013192	2	SM	NoDel	M1
BGE013201	3	SM	NoDel	M1
BGE013215	3	SM	NoDel	M1
BGE013218	4	SM	Del	M1
BGE013758	1	SM	NoDel	M1
BGE013761	4	SM	Del	M2
BGE013770	2	SM	NoDel	
BGE013773	2	SM	NoDel	M1
BGE013784	2	SM	NoDel	M2
BGE013795	4	SM	Del	M1
BGE015377	1	SM	NoDel	M1
BGE015390	2	SM	Del	M1
BGE015396	1	SM	Del	M1
BGE015397	1	SM	Del	M2
BGE015400	3	SM	NoDel	M2
BGE017171	2	LM	NoDel	
BGE018199	2	LM	NoDel	M1
BGE018205	2	SM	NoDel	
BGE018207	2	SM	NoDel	M1

BGE	Pop	GS1A	GS2A	GS2D
BGE018209	2	LM	NoDel	M1
BGE018213	3	SM	NoDel	M1
BGE018215	2	SM	NoDel	
BGE018228	4	SM	Del	M1
BGE018239	3	SM	NoDel	M1
BGE018241	3	SM	NoDel	M1
BGE018248	3	SM		M1
BGE018249	4	SM	Del	M1
BGE018258	4	SM	Del	M1
BGE018907	2	LM		M1
BGE018918	2	SM		M1
BGE023724	2	SM	Del	M1
BGE024864	2	SM	NoDel	M1
BGE024865	2	SM	Del	M1
BGE024867	2	SM	NoDel	M2
BGE024868	1	LM	Del	M1
BGE024869	2	LM	NoDel	M1
BGE024871	4	SM		M1
BGE025410	2	LM	Del	M1
BGE025413	2	SM	NoDel	
BGE029098	4	SM	Del	M1
BGE029796	4	SM	Del	
BGE030916	2	SM	NoDel	M1
BGE030918	2	SM	NoDel	
BGE036373	4	SM	Del	M1
BGE012591	1	SM	NoDel	M1
