# Supplementary material for: Development and marker-trait relationships of functional markers for glutamine synthetase GS1 and GS2 homoeogenes in bread wheat
Source: Mol Breed. 2023 Jan 19;43(2):8. doi: 10.1007/s11032-022-01354-0 (PMC10248667; doi:10.1007/s11032-022-01354-0)
Supplement: Supplementary file 11 — Supplementary file11 (PDF 63.8 KB) [file 11032_2022_1354_MOESM11_ESM.pdf]

Title: Development and marker-trait relationships of functional markers for glutamine synthetase *GS1* and *GS2* homoeogenes in bread wheat
Journal: Molecular Breeding
Authors: Pascual L. Solé-Medina A. Faci I. Giraldo P. Ruiz M and Benavente E.
Corresponding author: E. Benavente; Department of Biotechnology-Plant Biology. Universidad Politécnica de Madrid. Madrid. Spain; e.benavente@upm.es

Online Resource 11. *P* values of the F statistics from the analysis of variance of traits evaluated in the 187 bread wheat landraces forming the diversity panel (based on the phenotypic data available in López-Fernández et al. 2021). None of the GS gene-by-environment interactions was significant.

Source of variation	TKW	KS	GPC	SVol
GS1A	ns (1)	<0.0001	<0.0001	ns
GS2A	<0.0001	ns	<0.0001	ns
GS2D	0.0001	0.0102	0.0038	ns
Environment	<0.0001	<0.0001	<0.0001	<0.0001
GS1A × GS2A	0.0030	ns	ns	ns (1)
GS1A × GS2D	0.0019	0.0051	ns	ns
GS2A × GS2D	ns	ns	0.0187	ns

TKW: thousand-kernel weight; KS: kernels per spike; GPC: grain protein content; SVol: SDS-sedimentation volume.

ns= $P > 0.05$; (1) Means in genotypes with different marker alleles or non-allelic combinations are significantly different at $P = 0.05$.
